# An Evaluation of 90Y Bremsstrahlung SPECT Image Quality in the Presence of 99mTc: A Technical Perspective on Same-Day Radioembolization

**DOI:** 10.3390/curroncol31120554

**Published:** 2024-11-26

**Authors:** Grace Keane, Rob van Rooij, Marnix Lam, Arthur Braat, Maarten Smits, Hugo de Jong

**Affiliations:** Department of Radiology and Nuclear Medicine, University Medical Center Utrecht, 3584 CX Utrecht, The Netherlandsa.j.a.t.braat@umcutrecht.nl (A.B.);

**Keywords:** Yttrium, Technetium, post-treatment imaging, dosimetry, same-day radioembolization

## Abstract

In same-day radioembolization, 99mTc-MAA SPECT/CT, 90Y radioembolization, and post-treatment 90Y SPECT/CT procedures are conducted on the same-day, resulting in a dual-isotope environment of 90Y and 99mTc during post-treatment imaging. This study aimed to quantify the impact of 99mTc on 90Y bremsstrahlung-SPECT/CT image quality and to establish an optimised imaging protocol for both clinical practice, and with advanced reconstruction techniques. Utilising a NEMA IQ phantom, contrast recovery coefficients (CRCs) were measured to evaluate the 90Y image quality degradation caused by 99mTc. SPECT/CT scans of 90Y-only and 90Y with varying amounts of 99mTc were conducted using a standard protocol (90–120 keV energy window, high-energy collimator) and various dual-isotope protocols. The standard protocol resulted in a marked CRC reduction, with the largest sphere’s CRC decreasing from 0.21 (90Y-only) to 0.05 when 99mTc activity was 5% of 90Y. For an optimised protocol (160–200 keV energy window, high-energy collimator) CRC values were 0.16 for 90Y-only and 0.15 for 90Y+99mTc. The highest CRC values were achieved with an advanced Monte Carlo-based reconstruction, showing 0.58 for 90Y-only and 0.46 for 90Y+99mTc. Image quality degradation was noted in dual-isotope settings even when using an optimised protocol. Advanced reconstruction techniques markedly improved post-treatment image quality.

## 1. Introduction

Radioembolization or selective internal radiation therapy (SIRT) with yttrium-90 (90Y) is a well-established locoregional treatment option for primary and metastatic liver cancers [1]. The standard treatment pathway involves two key stages: an initial planning session with angiographic mapping and 99mTc-MAA scintigraphy, followed by a therapy session for 90Y microsphere administration and post-treatment imaging. In current practice, the treatment planning components of the workflow are conducted 1–2 weeks prior to the radioembolization treatment. This workflow has become the standard of care [2] for 90Y radioembolization; however, for patients with rapidly progressive disease or those travelling long distances, there is a clear benefit in expediting the treatment pathway. A growing body of literature demonstrates increased cost savings and improved treatment access by adopting same-day radioembolization [3,4,5]. In this approach, all procedures are amalgamated into one patient encounter performed on the same day.

Several studies have reported on a proof of concept for same-day SIRT [4,5], demonstrating that it is feasible for a selected patient population. However, there is a lack of literature evaluating same-day SIRT from a technical perspective and, specifically, the impact of this protocol on post-treatment imaging. Post-treatment imaging is regarded as an essential step in the 90Y treatment pathway in best practice guidelines [2,6], and several studies have demonstrated the association between post-treatment 90Y imaging-derived tumour-absorbed doses and outcome (e.g., response, survival) [7,8].

Post-treatment imaging can be conducted with SPECT/CT via imaging of bremsstrahlung photons or with PET/CT via imaging of annihilation photons. PET/CT is associated with greater image resolution and contrast than 90Y SPECT; however, due to the low positron branching ratio of 90Y [9], PET images can be noisy. SPECT/CT or SPECT was the modality of choice for post-treatment imaging in the majority of centres in a 2024 [10] survey of interventional radiologists. Due to the prevalence of SPECT in clinical centres globally, it is reasonable to assume that SPECT scanning will remain a mainstay of radioembolization treatment for the foreseeable future. There are some downsides associated with SPECT for post-treatment imaging, primarily the lack of clinically available scatter correction for 90Y SPECT, since energy window-based methods are not feasible with the continuous bremsstrahlung energy spectrum [11] (although, alternative methods using background compensation have been investigated as a form of quasi-scatter correction [12]). Still, the majority of sites depend on SPECT for 90Y imaging in the radioembolization pathway.

In the same-day SIRT approach, conducting pre-treatment and 90Y procedures in close succession gives rise to a dual-isotope environment, where both 99mTc and 90Y are present in the patient simultaneously at the time of the post-treatment assessment. In this scenario, even though during 90Y imaging the 99mTc activity is only a few percent of the 90Y activity, photon crosstalk between isotopes could reduce the image quality of 90Y bremsstrahlung SPECT scans, and consequently, the post-treatment visual assessment of the therapeutic activity distribution. This is due to overlapping spectra and the low bremsstrahlung yield. Although dual-isotope SPECT is a well-researched field with successful combinations including 99mTc with Thallium-201 or Iodine-123, these are isotopes with a clear photopeak, allowing for a window-based correction of photon crosstalk [13,14,15]. The lack of a photopeak in 90Y complicates post-treatment imaging in the same-day SIRT setting.

The purpose of this phantom study was twofold: firstly, to evaluate the influence of 99mTc on 90Y SPECT, and secondly, to determine the optimal imaging parameters to preserve 90Y SPECT image quality in the presence of 99mTc in dual-isotope protocols applicable both in clinical settings (with broad applicability on commercial imaging systems) and beyond (utilizing an advanced reconstruction algorithm).

## 2. Materials and Methods

Phantom images were used to identify 90Y signal degradation due to 99mTc via the measurement of contrast recovery coefficient (CRC). Imaging parameters that minimised 99mTc impact and optimised contrast recovery were identified.

### 2.1. Phantom Preparation

A phantom complying with the NEMA IEC NU2 2007 standards [16] for assessing image quality was adopted for this study. This phantom contains six coplanar spheres of increasing diameter (10 mm, 13 mm, 17 mm, 22 mm, 28 mm, and 37 mm) in a fillable torso-shaped background compartment and a cylindrical lung insert. The spherical inserts of the phantom were filled with 90Y chloride of activity concentration 2.37 MBq/mL and the background volume with a concentration of 0.30 MBq/mL, which resulted in an approximate 8:1 ratio. This ratio is higher than typical tumour-to-normal tissue ratios in clinical scenarios [17,18] but was chosen for this investigation due to its prior use in quantitative 90Y imaging assessments [19] and alignment with the recommendations of NEMA Standards Publication NU 1-2023 [20]. A total of six experiments were conducted using high-energy (HE) and medium-energy (ME) collimators and different 90Y/99mTc activity ratios. Each experimental configuration was acquired using three different energy windows (90–120 keV, 160–200 keV, and 200–250 keV) (Table 1). 90Y-only scans were conducted in experiments 1 and 2, and following this, 99mTc was added to the background of the phantom, and a further 4 acquisitions (experiments 3–6) were performed. 99mTc was added only to the background compartment of the phantom and not the spheres to avoid the unintentional increase in the measured contrast recovery in 90Y reconstructions due to the 99mTc photons recorded in the 90Y energy window, and therefore introducing bias in the 90Y image quality metrics. In experiments 5 and 6, scan time was prolonged in order to obtain equivalent count statistics as experiments 1–4. The resulting effective activities (Aeff) are given in Table 1. Over the course of the measurements, the effective 90Y activity in the phantom remained approximately 3 GBq. For each experiment, repetitions were acquired to calculate an average and evaluate the standard deviation.

The activities were chosen to approximate the highest clinically relevant 99mTc percentage (5%) that could be expected in a same-day SIRT patient at the time of post-treatment imaging, and increasingly elevated ratios to test the robustness of the 90Y-99mTc dual-isotope protocols. Guidelines on pre-treatment imaging indicate 150 MBq (±20%) of 99mTc MAA be administered for the treatment planning scintigraphy study and scanning be performed as soon as possible after administration [2]. Various values for mean same-day SIRT procedure time (defined as the period from the start of the treatment planning angiography to the end of the radioembolization procedure) are reported in the literature, ranging from 3 h to 7 h [3,4,5]. A standard vial size of 90Y is 3 GBq (±10%). A 5% 99mTc level represents the percentage that would result assuming an injection of 180 MBq of 99mTc, a 3 h time interval, and 2.7 GBq of 90Y.

### 2.2. Imaging

Imaging was performed on a Symbia Intevo scanner (Siemens, Erlangen, Germany). SPECT/CT scans of 90Y-only and 90Y with varying amounts of 99mTc were acquired using a local site protocol (developed considering only single-isotope 90Y bremsstrahlung SPECT) and a number of specifically designed 90Y-99mTc dual-isotope protocols.

The local site protocol acquisition parameters included an energy window of 90–120 keV with high-energy collimators, and reconstruction parameters included iterative reconstruction in the form of ordered subset expectation maximisation (OSEM) with 10 iterations, 8 subsets (Flash 3D, Siemens, Erlangen, Germany), and 5 mm full-width half-maximum Gaussian post-filtering. Whilst this specific combination of imaging and reconstruction parameters are not explicitly reported elsewhere in the literature, the local site protocol was deemed adequate for this investigation, as there is no standardised protocol for 90Y imaging, and it bears many similarities to previously reported protocols [2,12].

**Figure 1:**
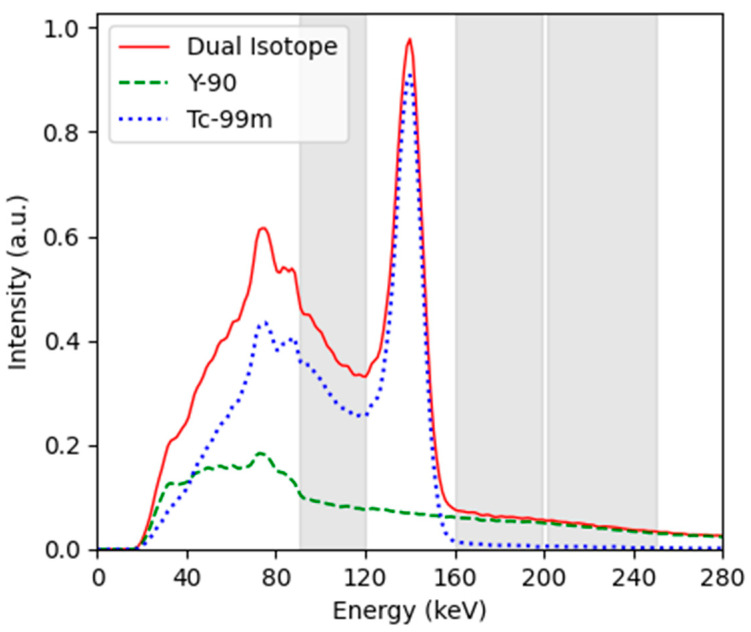
Measured energy spectrum. The solid red line is the dual-isotope spectrum recorded when both 90Y and (13%) 99mTc were present, the green dashed line is the 90Y spectrum, and the blue dashed line is the 99mTc spectrum (created via subtraction of the 90Y from the dual-isotope spectrum). The 3 energy windows used in this investigation were 90–120 keV, 160–200 keV, and 200–250 (highlighted in grey).

The 90Y-99mTc dual-isotope protocols also used OSEM with 10 iterations and 8 subsets, and either high-energy (HE) or medium-energy (ME) collimators to discriminate the high-energy photons in the bremsstrahlung spectrum. The energy windows utilised (160–200 keV and 200–250 keV) were selected so as to capture regions of the 90Y bremsstrahlung spectrum minimally impacted by 99mTc. The spectra for 90Y and 99mTc are shown in Figure 1 together with the dual-isotope spectrum and the acquired windows. The 160–200 keV window still has a small contribution from 99mTc counts (which may be visualised by the tail in the 99mTc spectrum in Figure 1), but benefits from capturing the higher count intensity of 90Y. The energy window of 200–250 keV should have a negligible contribution from 99mTc, but at this energy, the 90Y bremsstrahlung also begins to tail off. It is clear that, if the standard energy window is used (90–120 keV), there will be considerable contamination from 99mTc.

Finally, images acquired via the local site protocol and 90Y-99mTc dual-isotope protocols were reconstructed using the Utrecht Monte Carlo system (UMCS), an iterative reconstruction algorithm that incorporates Monte Carlo-based scatter correction, attenuation correction, and modelling the collimator detector response (CDR) (fully described elsewhere [21,22,23]). For the purpose of this work, UMCS was adapted for 90Y for the selected windows: 90–120 keV, 160–200 keV, and 200–250 keV.

### 2.3. Image Quality Analysis

Image quality was assessed via measurements of contrast recovery. Spherical VOIs matching the known sphere volume were placed over the corresponding spheres using an in-house-built script. The VOIs were separately defined on a high-quality CT scan, and then registered to each of the CTs of the SPECT/CT scans using Elastix 5.2.0 [24,25]. A 3D annulus VOI (inner radius = 39 mm; outer radius = 76 mm; and length = 50 mm) was placed in the background in the space surrounding the lung insert on slices adjacent to those containing the spheres and extended down the longitudinal length of the phantom. CRCs were calculated by dividing the measured contrasts by the nominal, true contrasts determined from the reported injected activities as shown in Equation (1).

(1) CRC=CSCB−1R−1

where *C_S_* is the measured mean signal in the spherical VOI, *C_B_* is the measured mean signal in the background, and *R* is the sphere-to-background activity concentration ratio. *CRC* was calculated for each repetition, and an average was determined, together with the standard deviation.

The image quality degradation of 90Y bremsstrahlung SPECT resulting from imaging in a 99mTc environment was quantified as the difference in CRC between scans of 90Y-only and 90Y+99mTc. In all instances, CRC was plotted for the largest 4 spheres, since partial volume effect and noise dominated for the smaller sphere measurements.

A flowchart depicting the study design is shown in Supplemental Figure S1.

## 3. Results

### 3.1. Impact of 99mTc on 90Y Bremsstrahlung SPECT/CT with Local Site Protocol

**Figure 2:**
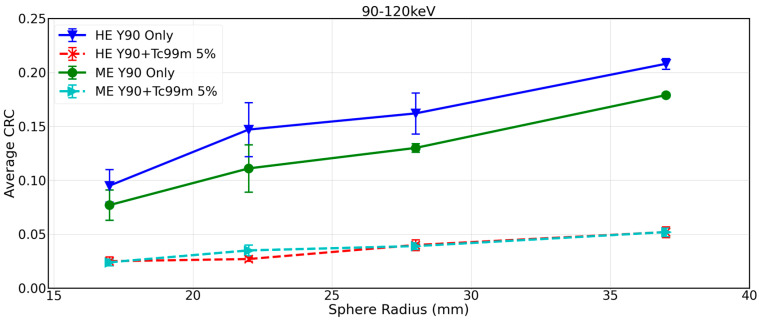
CRC as a function of sphere diameter for 90Y and 90Y+99mTc at approximately 5% 99mTc, with 90–120 keV energy window, HE collimators (local site protocol), and 90–120 keV energy window, ME collimators.

Figure 2 shows CRCs by sphere size for 90Y-only (experiments 1 and 2, Table 1) and 90Y+99mTc SPECT with approximately 5% 99mTc (experiments 5 and 6, Table 1) reconstructed with the local site protocol. Significant reduction in CRC is evident across all sphere sizes in the 90Y+99mTc phantom. As an example, for the local site protocol (90–120 keV, HE), CRC dropped from 0.21 to 0.05 for the largest sphere (37 mm diameter). This demonstrates the inadequacy of the local site protocol for imaging 90Y in the presence of 99mTc.

**Figure 3:**
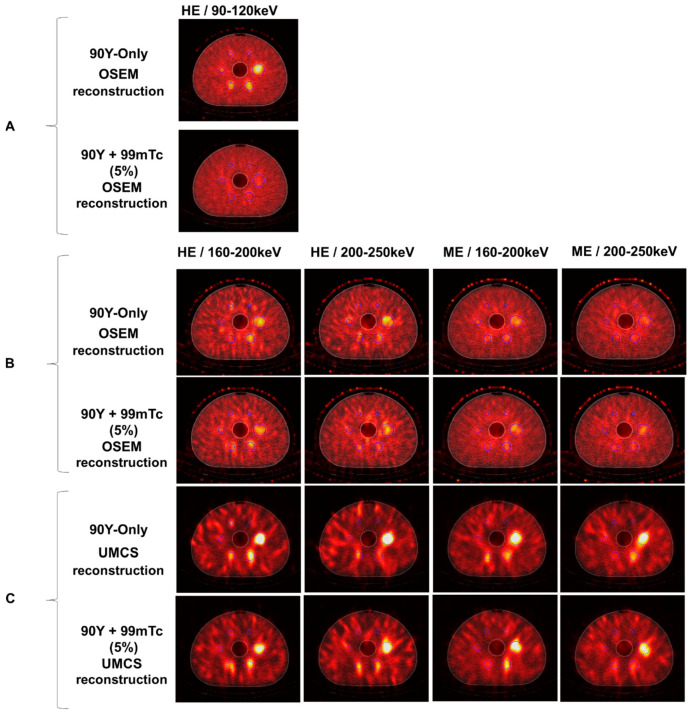
An axial slice through the 90Y-only and 90Y+99mTc phantom at approximately 5% 99mTc: (**A**) the results of measurement with the local site protocol, (**B**) the results of measurement with the dual-isotope protocols and OSEM reconstruction, and (**C**) the results of measurement with the dual-isotope protocols and UMCS reconstruction.

An examination of a central transverse slice through the SPECT volume where all spheres were visible in the co-registered CT demonstrated visible reduction in contrast in 90Y+99mTc phantom as compared to 90Y alone when using the local site protocol (90–120 keV, HE) (Figure 3A).

### 3.2. Impact of 99mTc on 90Y Bremsstrahlung SPECT/CT with Same-Day SIRT Protocols Applicable to Commercial Imaging Systems

**Figure 4:**
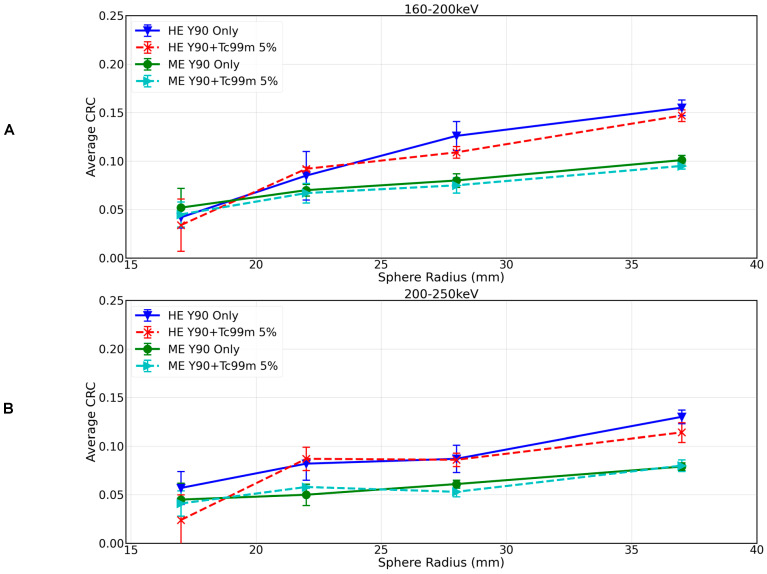
CRC as a function of sphere diameter for 90Y-only and 90Y+99mTc at approximately 5% 99mTc for (**A**) the 160–200 keV HE and ME protocols reconstructed with OSEM and (**B**) 200–250 keV HE and ME protocols reconstructed with OSEM.

Figure 4 shows CRC by sphere size for the 90Y-99mTc dual-isotope protocols with energy windows 160–200 keV with HE and ME collimators (Figure 4A) and 200–250 keV with HE and ME, respectively (Figure 4B) (experiment 5 and 6 in Table 1), reconstructed with OSEM. For the 90Y+99mTc measurements, the 99mTc activity concentration was 5%(ME) or 6%(HE). There was minimal difference in 90Y-only and 90Y+99mTc when using the 160–200 keV/HE protocol (CRC of 0.16 and 0.15 for the 37 mm sphere), and the difference was further reduced when using the 160–200 keV/ME protocol (CRC of 0.10 in both instances for the 37 mm sphere). The 200–250 keV/HE protocol resulted in a CRC for the 37 mm sphere of 0.13 for 90Y-only and 0.11 for 99mTc+90Y (see Figure 4), and for the 200–250 keV/ME protocol, there was no difference in CRC between 90Y-only and 99mTc+90Y (CRC of 0.08 in both instances for the 37 mm sphere).

Examination of the central transverse slice for the different imaging protocols for 90Y-only and 90Y+99mTc (approximately 5% 99mTc) demonstrated that, as the energy of the window increased, the image quality decreased (and consequently, so did the corresponding CRC). Image quality appeared improved with the HE collimators vs. ME collimators (Figure 3B).

Comparing the best image quality for 90Y only (derived via the local site protocol, Figure 2) with the best image quality that can be achieved in the dual-isotope setting (derived via the 160–200 keV/HE protocol, Figure 4A), image quality was superior in the 90Y-only image, and the CRC for the largest sphere being 0.21 for 90Y only and 0.15 for 99mTc+90Y (29% reduction).

**Figure 5:**
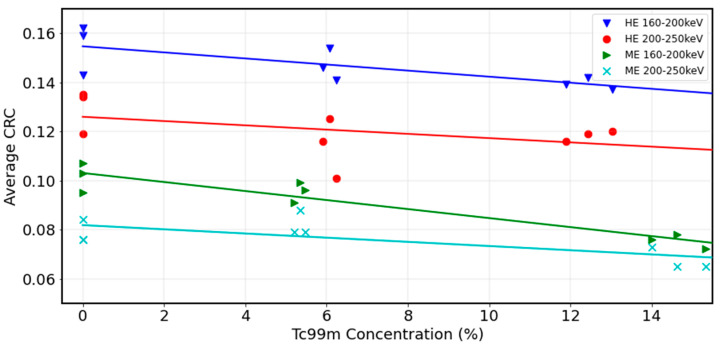
CRC vs. 99mTc activity concentrations for the 37 mm sphere in NEMA IQ phantom.

Contrast recovery was also plotted against 99mTc activity concentration for the range of activities using different 90Y+99mTc dual-isotope protocols (experiments 1–6 in Table 1). Figure 5 shows the largest sphere diameter at 0%, approximately 5% and approximately 14% 99mTc. The impact of 99mTc contamination is most visible in the 160–200 keV window for both collimators where CRC reduces incrementally with increasing 99mTc from 0% to 14%. CRC is less hampered by 99mTc in the 200–250 keV window; however, CRC is notably diminished in this window as compared to the other windows. These results would suggest that the 160–200 keV/HE is a robust protocol since CRC is optimised on this window, and the impact of 99mTc contamination is not substantial over the clinically relevant 99mTc activity concentration range.

### 3.3. Impact of 99mTc on 90Y Bremsstrahlung SPECT/CT with Same-Day SIRT Protocols Including Advanced Reconstruction

**Figure 6:**
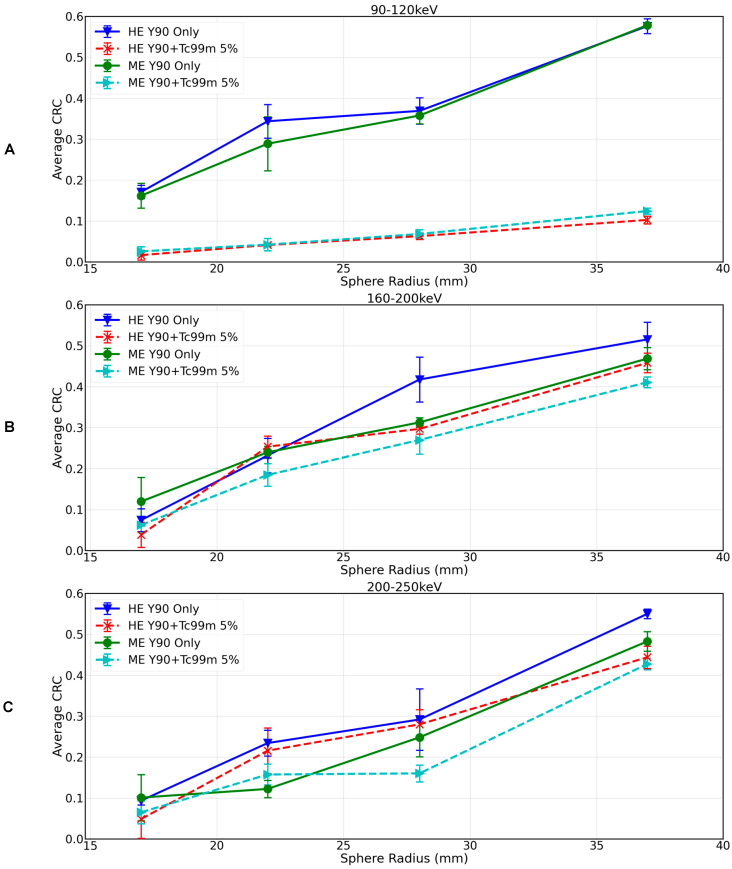
CRC as a function of sphere diameter for 90Y-only and 90Y+99mTc at approximately 5% 99mTc for (**A**) the 90–120 keV window with HE and ME collimators, (**B**) the 160–200 keV window with HE and ME collimators, and (**C**) the 200–250 keV window with HE and ME collimators, all reconstructed with the UMCS reconstruction protocol.

Figure 6 shows CRC by sphere size for 90Y-only (experiments 1 and 2, Table 1) and 90Y+99mTc SPECT with approximately 5% 99mTc (experiments 5 and 6, Table 1) reconstructed with the UMCS protocol. For all spheres, the UMCS reconstructed images showed improved contrast recovery as compared to the standard local site reconstruction. For example, for 90Y only, the CRC for the 37 mm sphere measured on local site acquisition and reconstruction protocol (Figure 2) vs. the local site protocol with UMCS reconstruction (Figure 6A) increased from 0.21 to 0.58. Similarly, for Y90+99mTc, the CRC for the 37 mm sphere as measured on the dual-isotope protocols, 160–200 keV/HE and 160–200 keV/ME, with local site reconstruction (Figure 4A) vs. UMCS reconstruction (Figure 6B), increased from 0.15 to 0.46 and 0.10 to 0.41, respectively. For 200–250 keV/HE and 200–250 keV/ME, the CRC for the 37 mm sphere with local site reconstruction (Figure 4B) vs. UMCS reconstruction (Figure 6C) increased from 0.11 to 0.44 and 0.08 to 0.43, respectively.

Comparing the best image quality for 90Y-only (derived via the local site acquisition protocol and UMCS reconstruction protocol, Figure 6A) with the best image quality that can be achieved in the dual-isotope setting (derived via the 160–200 keV/HE acquisition and UMCS reconstruction protocol, Figure 6B), image quality was superior in the 90Y-only image, with the CRC for the largest sphere being 0.58 for 90Y-only and 0.46 for 99mTc+90Y (20% reduction).

Examination of the central transverse slice for 90Y-only and 90Y+99mTc demonstrated that contrast is better preserved in the 90Y+99mTc setting when UMCS is implemented (Figure 3C).

## 4. Discussion

Post-treatment images using SPECT/CT or PET/CT are routinely used to verify dose delivery and microsphere distribution after 90Y SIRT and, although less well established, to estimate the post-treatment absorbed dose in tumours and normal tissue, which represent important indicators for the efficacy and toxicity of the treatment. Since many centres do not have access to PET/CT, bremsstrahlung SPECT/CT was considered in this study. In the context of a same-day SIRT treatment, it is essential that image quality of post-treatment SPECT/CT images, and consequently, confidence in visual assessment of the 90Y activity distribution, is not degraded excessively by presence of 99mTc. The objective of this study was twofold: firstly, to evaluate the impact of 99mTc on post-treatment Y-90 bremsstrahlung SPECT/CT scans, and secondly, to determine the optimal imaging parameters to preserve 90Y SPECT image quality in the presence of 99mTc in dual-isotope protocols applicable both in clinical settings (with broad applicability on commercial imaging systems) and beyond (utilizing an advanced reconstruction algorithm).

Regarding the first objective, these results demonstrated that, when no conditions were imposed on the imaging protocol to reflect the presence of 99mTc, image quality was significantly impacted. As an example, using the local site acquisition and reconstruction protocol, the CRC for the 37 mm NEMA phantom sphere deteriorated from 0.21 to 0.05 for 90Y-only and 90Y+99mTc, respectively. It is reasonable to assume that, in a patient scenario, clinically relevant features of the distribution could be obscured. This result is understandable given the positioning of the energy window in the local site protocol relative to the 99mTc photopeak; therefore, in order to make same-day radioembolization post-treatment imaging a viable option, a specific protocol designed to mitigate the effect of 99mTc is required.

Regarding the second objective, several technical recommendations on acquiring and reconstructing 90Y bremsstrahlung SPECT/CT scans for the purpose of post-treatment dosimetry have been published, and in general, optimisation is focused on choice of collimators, image matrix size, reconstruction parameters, and energy windows. In this study, we evaluated the role of energy window, collimator choice, and reconstruction parameters in both isolation of the 90Y signal and optimisation of image quality.

In a dual-isotope 99mTc and 90Y setting, there are two competing effects at play: lower energy windows benefit from capturing maximal bremsstrahlung signal, and higher energy windows more effectively exclude the 99mTc signal and therefore result in the smallest variance between 90Y and 99mTc+90Y. Finding the right balance is key. It was demonstrated that, using a standard window, the 99mTc overwhelms the 90Y bremsstrahlung and therefore must be excluded; however, too high an energy window and the measured signal is so low that images are effectively non-diagnostic. Results indicated an energy window of 160–200 keV to be the best compromise to mitigate these competing effects, since contrast recovery was optimised and the impact of 99mTc was minimal.

An important consideration in image quality optimisation is maximising resolution. Typically, ME collimators are selected for this purpose; however, in our dual-isotope protocol, HE collimators were chosen despite being associated with lower resolution. It was evident for all experiments that HE collimators resulted in a higher CRC. The thicker septa of the HE collimators more effectively prevented septal penetration, consequently suppressing the background signal, which had a strong positive effect on contrast recovery.

Previous studies [17] have demonstrated that reconstruction including Monte Carlo-based compensation for scatter, attenuation effects, and energy-dependent modelling of the collimator detector response can effectuate considerable improvement in 90Y Bremsstrahlung SPECT image quality. This study investigated whether the UMCS fast Monte Carlo simulator could improve image quality in a dual-isotope setting. The technique facilitated a more complete isolation of the Y90 signal, as evidenced by the relative reduction in CRC between 90Y-only and 90Y+99mTc (20% for the 37 mm sphere) when using the UMCS reconstruction, which was lower than that observed when using the dual-isotope protocol with OSEM reconstruction (29% for the 37 mm sphere). UMCS reconstructions were also associated with a notable increase in contrast recovery, and the maximum CRC achieved for the 37 mm sphere for 90Y+99mTc using UMCS was 0.46 vs. 0.15 for the local site protocol.

International guidelines advise against using commercially available 90Y bremsstrahlung SPECT and associated reconstruction algorithms for quantitative assessment and absorbed dose computation [2]. Although specific corrections and advanced reconstruction algorithms can potentially enhance SPECT image quality (such as the UCMS reconstruction investigated in this study), these approaches are not widely accessible in clinical settings. The challenges with 90Y bremsstrahlung SPECT in terms of quantitative accuracy are further compounded in the context of dual-isotope procedures. Therefore, the suitability of 90Y bremsstrahlung SPECT scanning for this purpose, as commonly practised in most centres, warrants careful consideration.

Despite these limitations, an optimised protocol for dual-isotope scanning of 90Y with a 99mTc background that may be implemented on a commercially available imaging system was defined (160–200 keV/HE with OSEM reconstruction). It is possible to visually distinguish the 37 mm sphere from the background using images derived via the proposed dual-isotope protocol, and therefore, these images may be sufficient to give an indication of tumour targeting or for the purpose of delivery verification. In a treatment scenario, where visual assessment of activity distribution in the perfused volume is all that is required, these images could be considered suitable.

The image quality achievable from a dual-isotope acquisition protocol partnered with the UMCS reconstruction was markedly improved compared to what may be achieved on a commercially available imaging system, surpassing both 90Y bremsstrahlung SPECT using a local site protocol and 90Y+99mTc using dual-isotope protocols with OSEM reconstruction. The maximum CRC achieved for the 37 mm sphere (0.5) in the 90Y+99mTc setting, when using 160–200 keV/HE with UMCS, is only marginally reduced compared to values reported for spheres of equivalent size in 90Y PET (0.6–0.8 [26,27]), an established modality for 90Y dosimetry. This suggests that 90Y+99mTc SPECT/CT images may be of sufficient quality to enable dosimetric applications if an optimised dual-isotope acquisition protocol is used and reconstruction is performed with appropriate compensation models. At a minimum, these images facilitate a more effective qualitative assessment of 90Y activity distribution following same-day SIRT.

This study has limitations. Firstly we used a clinical reconstruction protocol without background compensation, but it is known that background compensation can substantially improve CRC. Siman et al. [12] demonstrated compensating for bremsstrahlung background improved recovery coefficient from 39% to 90% on a 37 mm spherical insert. Also, an examination of what image quality reduction is clinically acceptable was beyond the scope of this preliminary phantom study. Since this study exclusively focused on a NEMA phantom where the spherical inserts were taken to represent tumours, additional research with an anthropomorphic phantom capable of modelling a radiation segmentectomy activity distribution (i.e., with larger fillable compartments) would be of interest, as radiation segmentectomy treatment scenario is often paired with a same-day workflow. Finally, in a clinical context, it is expected that the 99mTc and 90Y activity distributions will be comparable. This phantom set-up is therefore not clinically representative due to the disparity between the two activity distributions; however, it facilitated an unbiased measurement of 90Y signal degradation due to 99mTc.

## 5. Conclusions

In the context of a same-day SIRT procedure, it is important that image quality of post-treatment 90Y imaging is maintained. Optimised parameters for same-day SIRT imaging have been reported in a dual-isotope protocol applicable for commercial imaging systems; however, even when using this optimised protocol, some degree of bremsstrahlung SPECT/CT image quality degradation is to be expected in a dual-isotope setting. Caution is therefore required while reviewing post-treatment same-day SIRT images for centres using SPECT/CT as their imaging modality. An advanced reconstruction algorithm including Monte Carlo-based compensation for scatter, attenuation, and modelling of the CDR markedly improved image quality and could therefore facilitate a more effective qualitative assessment of 90Y activity distribution following same-day SIRT.

## Figures and Tables

**Table 1:** Schedule of phantom experiments, showing the radionuclide, imaging parameters, and starting activities for the given measurement.

Exp.	Scan Time (min)	Collimator	[A^eff^] 90Y (GBq)	[A^eff^] 99mTc (MBq)	% 99mTc
1 90Y only	20 (3 reps *)	HE	3.2	0	0%
2 90Y only	20 (3 reps)	ME	3.1	0	0%
3 90Y+99mTc	20 (3 reps)	ME	3.1	450	15%
4 90Y+99mTc	20 (3 reps)	HE	3.0	376	13%
5 90Y+99mTc	25 (3 reps)	HE	3.1	189	6%
6 90Y+99mTc	25 (3 reps)	ME	3.1	165	5%

* reps = repetitions. NB. For each repetition, in experiments 3–6, the three energy windows (90–120 keV, 160–200 keV, and 200–250 keV) were acquired simultaneously. These acquisitions were reconstructed using the local site reconstruction protocol and advanced reconstruction protocol.

## Data Availability

Data available upon reasonable request.

## Supplementary Materials

The following supporting information can be downloaded at: https://www.mdpi.com/article/10.3390/curroncol31120554/s1, Figure S1: Flowchart of study design.
