# Supplementary figures and images for: An Evaluation of 90Y Bremsstrahlung SPECT Image Quality in the Presence of 99mTc: A Technical Perspective on Same-Day Radioembolization
Source: Curr Oncol. 2024 Nov 26;31(12):7511–22. doi: 10.3390/curroncol31120554 (PMC11674725; doi:10.3390/curroncol31120554)

## Supplemental Figures

Figure S1. Flowchart of study design

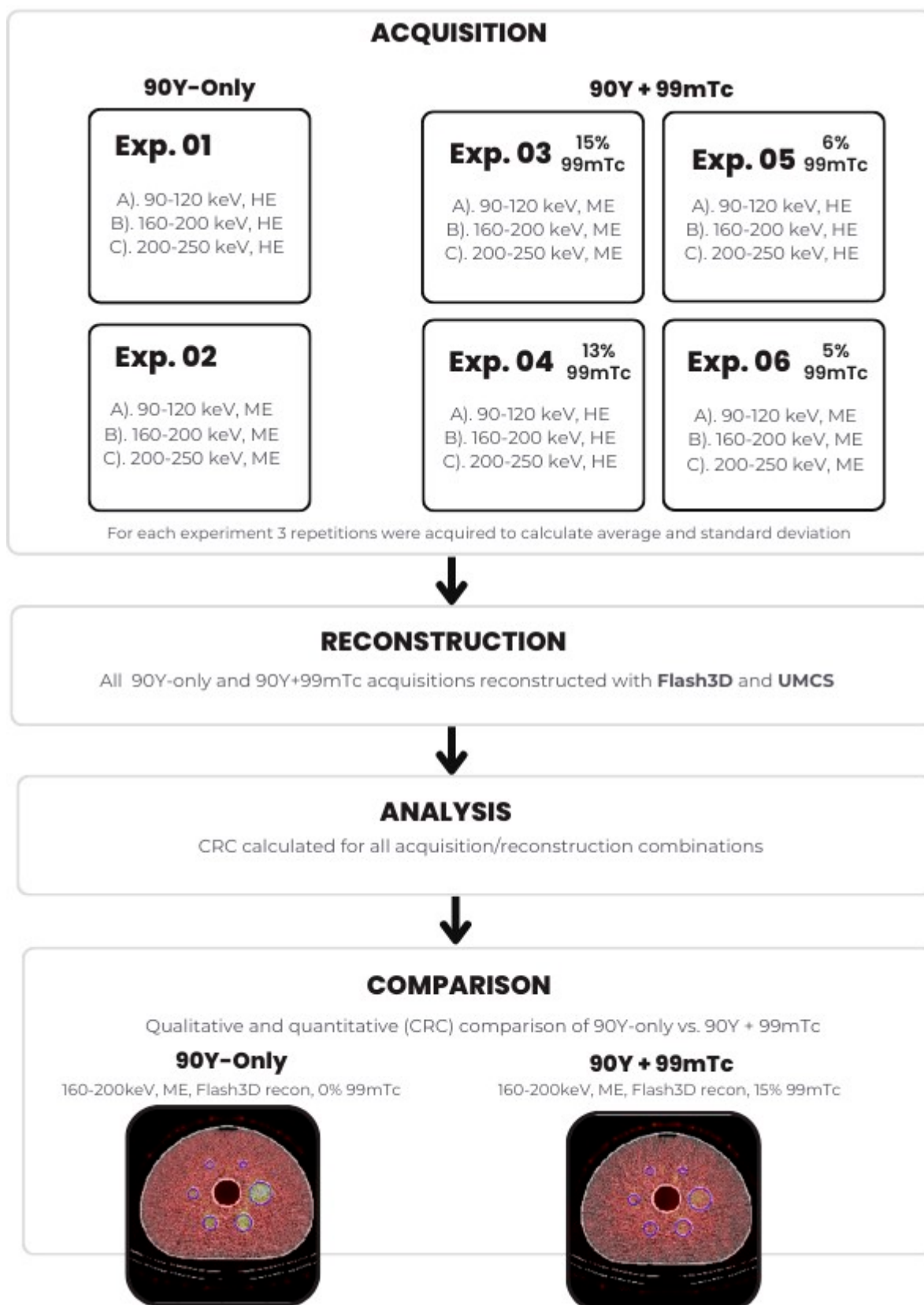

Supplement: Supplementary file 1 [file curroncol-31-00554-s001.zip › curroncol-3186360-supplementary.pdf]
